# Evaluating the effectiveness of retention forestry to enhance biodiversity in production forests of Central Europe using an interdisciplinary, multi‐scale approach

**DOI:** 10.1002/ece3.6003

**Published:** 2020-01-14

**Authors:** Ilse Storch, Johannes Penner, Thomas Asbeck, Marco Basile, Jürgen Bauhus, Veronika Braunisch, Carsten F. Dormann, Julian Frey, Stefanie Gärtner, Marc Hanewinkel, Barbara Koch, Alexandra‐Maria Klein, Thomas Kuss, Michael Pregernig, Patrick Pyttel, Albert Reif, Michael Scherer‐Lorenzen, Gernot Segelbacher, Ulrich Schraml, Michael Staab, Georg Winkel, Rasoul Yousefpour

**Affiliations:** ^1^ Chair of Wildlife Ecology and Management Faculty of Environment and Natural Resources University of Freiburg Freiburg Germany; ^2^ Chair of Silviculture Faculty of Environment and Natural Resources University of Freiburg Freiburg Germany; ^3^ Forest Research Institute of Baden‐Württemberg (FVA) Freiburg Germany; ^4^ Conservation Biology Institute of Ecology and Evolution University of Bern Bern Switzerland; ^5^ Biometry and Environmental System Analysis Faculty of Environment and Natural Resources University of Freiburg Freiburg Germany; ^6^ Chair of Remote Sensing and Landscape Information Systems Faculty of Environment and Natural Resources University of Freiburg Freiburg Germany; ^7^ Black Forest National Park Bad Peterstal‐Griesbach Germany; ^8^ Chair of Forestry Economics and Forest Planning Faculty of Environment and Natural Resources University of Freiburg Freiburg Germany; ^9^ Chair of Nature Conservation and Landscape Ecology Faculty of Environment and Natural Resources University of Freiburg Freiburg Germany; ^10^ Chair of Sustainability Governance Faculty of Environment and Natural Resources University of Freiburg Freiburg Germany; ^11^ Chair of Site Classification and Vegetation Science Faculty of Environment and Natural Resources University of Freiburg Freiburg Germany; ^12^ Geobotany Faculty of Biology Freiburg Germany; ^13^ Resilience Programme European Forest Institute Bonn Germany

**Keywords:** Black Forest, ConFoBi, deadwood, forest ownership, habitat tree, landscape, translational research

## Abstract

Retention forestry, which retains a portion of the original stand at the time of harvesting to maintain continuity of structural and compositional diversity, has been originally developed to mitigate the impacts of clear‐cutting. Retention of habitat trees and deadwood has since become common practice also in continuous‐cover forests of Central Europe. While the use of retention in these forests is plausible, the evidence base for its application is lacking, trade‐offs have not been quantified, it is not clear what support it receives from forest owners and other stakeholders and how it is best integrated into forest management practices. The Research Training Group ConFoBi (Conservation of Forest Biodiversity in Multiple‐use Landscapes of Central Europe) focusses on the effectiveness of retention forestry, combining ecological studies on forest biodiversity with social and economic studies of biodiversity conservation across multiple spatial scales. The aim of ConFoBi is to assess whether and how structural retention measures are appropriate for the conservation of forest biodiversity in uneven‐aged and selectively harvested continuous‐cover forests of temperate Europe. The study design is based on a pool of 135 plots (1 ha) distributed along gradients of forest connectivity and structure. The main objectives are (a) to investigate the effects of structural elements and landscape context on multiple taxa, including different trophic and functional groups, to evaluate the effectiveness of retention practices for biodiversity conservation; (b) to analyze how forest biodiversity conservation is perceived and practiced, and what costs and benefits it creates; and (c) to identify how biodiversity conservation can be effectively integrated in multi‐functional forest management. ConFoBi will quantify retention levels required across the landscape, as well as the socio‐economic prerequisites for their implementation by forest owners and managers. ConFoBi's research results will provide an evidence base for integrating biodiversity conservation into forest management in temperate forests.

## Graphical abstract

This paper describes the background, research questions and design of the Research Training Group “ConFoBi” (Conservation of Forest Biodiversity in Multiple‐use Landscapes of Central Europe). ConFoBi focusses on the effectiveness of retention forestry, that is, retaining structural elements of old forests within a production forest matrix, and combines ecological studies on forest biodiversity with social and economic studies of biodiversity conservation across multiple spatial scales. The aim of ConFoBi is to assess whether and how structural retention measures are appropriate for the conservation of forest biodiversity in multiple‐use landscapes of temperate Europe.

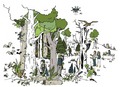

## 1 INTRODUCTION

### 1.1 Forest biodiversity and the need for integrated forestry

Since the UN Convention on Biological Diversity (CBD) was signed in 1992, the conservation of biodiversity has become a global commitment. Yet, species extinction rates continue to accelerate, as recently documented by the Intergovernmental Science‐Policy Platform on Biodiversity and Ecosystem Services (IPBES, 2019).

In Europe, forests are the dominant natural vegetation form, and present the primary evolutionary background of today's plant and animal species and communities. Forests provide habitat for numerous species and therefore play a key role in the conservation of biodiversity (European Environment Agency, 2016; Forests Europe, 2015).

Forests are also valued for safeguarding ecosystem services, such as production of wood, protection against natural disasters, and provision of recreational opportunities. Further, forests and forestry traditionally play an important role in European culture, provide employment, and contribute to local economies. In view of these manifold human demands for wood and other ecosystem goods and services, the vast majority of forests in Europe are managed to serve economic, social, and environmental functions at the same time (Forests Europe, 2015).

These multiple functions are not always easily reconciled (Niemelä et al., 2005; Verkerk et al., 2014). For example, temperate forests managed for high economic benefit are unlikely to be rich in biodiversity, because trees and stands are typically harvested at economic maturity, which can be reached, depending on the tree species, after 60–200 years. This is relatively early in the life span of forests whose trees may live for several hundred years, and long before the development of old and structurally rich forest successional stages with their high level of biodiversity and many unique species (Gustafsson et al., 2012; Hilmers et al., 2018; Scherzinger, 1996). Thus production forests, here understood as “forests available for wood supply” (FAWS) (UNECE/FAO, 2000) lack in many cases the structural heterogeneity of natural forest ecosystems. Specifically, they lack late successional stages and structural elements (e.g., snags, coarse woody debris, and canopy gaps) created by decay processes or natural disturbances. This is of major hindrance for various groups, such as epiphytic lichens and plants, saproxylic fungi and invertebrates, and cavity‐nesting mammals and birds, which depend on the specific resources available in old forests (Goldberg, Kirby, Hall, & Latham, 2007; Kraus & Krumm, 2013; Siitonen, Martikainen, Punttila, & Rauh, 2000). Forests rich in late successional stages and structural elements are extremely rare in Europe. Indeed, across Europe, only marginal proportions (1.5%) of forest are permanently taken out of production (“no active intervention,” Forests Europe, 2015, p. 160) and set aside in protected areas such as National Parks and other strictly protected forests. Reserves without human intervention are valuable reference areas for research on natural forest ecosystems, and important cornerstones for the conservation of forest biodiversity. However, a reserve‐based segregative approach alone cannot ensure the conservation of forest biodiversity because small reserve sizes and insufficient connectivity limit the effectiveness of these remaining islands of natural forest for biodiversity conservation (Bollmann & Braunisch, 2013).

Thus, integrative conservation measures in production forests are a crucial complement in safeguarding forest biodiversity not only at the local scale of protected areas, but in particular across entire landscapes. Increasingly, retention‐forestry approaches (Bauhus, Puettmann, & Messier, 2009) are applied “to provision for continuity in structural, functional, and compositional elements from the preharvest to the postharvest forest” (Gustafsson et al., 2012). Retention forestry, that is, retaining small patches or structural elements within a production forest matrix, is implemented particularly in public forests, and often related to requirements originating from EU legislation. Integration of biodiversity conservation into forest management is a major policy goal throughout Europe (cf. the EU Forest Strategy, European Commission, 2013; and the EU Biodiversity Strategy, European Commission, 2011; the German National Strategy on Biological Diversity, BMU (2007); and the German Forest Strategy 2020, BMELV, 2011). In the federal system of Germany, the responsibility for programme implemention is focused on state level (ForstBW, 2016).

### 1.2 State of knowledge and research gaps

Retention forestry has been practiced and extensively studied in clear‐cutting systems for 30 years (Fedrowitz et al., 2014; Gustafsson et al., 2012; Mönkkönnen, Ylisirniö, & Hämäläinen, 2009). However, there is still a need to extend research on the effectiveness of retaining structural elements for biodiversity conservation from clear‐cutting to the continuous‐cover forestry systems (Gustafsson et al., 2019), which dominate major parts of the temperate regions in Europe.

Few studies have examined retention approaches in selectively logged forests in the temperate zone (Müller, 2005); thus, retention guidelines, as they are currently implemented across central Europe, are based on plausibility and expert knowledge rather than on scientific evidence (Vítková, Bače, Kjučukov, & Svoboda, 2018). For example, in the state forests of Bavaria, Germany, an average of ten habitat trees per hectare has to be retained in near‐natural stands (BaySF, 2009), whereas in the neighboring state of Baden‐Württemberg, one group of habitat trees consisting of about 15 trees is to be secured per 3 ha (ForstBW, 2016). Across Europe, all countries use their own, variable prescriptions (Sotirov, 2017; Winter et al., 2014). This also holds for FSC and PEFC certification standards in Europe: In national certification standards, quantitative retention targets are either not specified, or they vary greatly (e.g., 1–10 living habitat trees per ha; 1–20 dead trees per ha) among European countries (Gustafsson et al., 2019).

Trade‐offs between conservation and production objectives may explain different thresholds set in conservation programmes in production forests. Yet, there is obviously also uncertainty about the thresholds required for retention amounts to achieve the desired effects on biodiversity. Recommendations to date, that is, for the required amount of deadwood and habitat trees, have been based mostly on single taxonomic groups such as forest birds, saproxylic insects, lichens, or fungi (Müller & Bütler, 2010; Sandström et al., 2019). Studies investigating a wide spectrum of species including multiple taxa and trophic groups in the same study system are rare (Franklin, Macdonald, & Nielsen, 2019; Müller & Bütler, 2010; Paillet et al., 2018; Ranius & Fahrig, 2006; Vítková et al., 2018). Further, relationships between forest structure and biodiversity have not been studied comprehensively in a landscape context (Mori, Tatsumi, & Gustafsson, 2017). Landscape ecology and community ecology theory (Leibold et al., 2004; Lindenmayer & Franklin, 2002; Tscharntke et al., 2012) for instance would predict that a network of stands rich in deadwood may contribute more to biodiversity than uniformly distributed retention of moderate amounts of deadwood across the landscape (Müller & Bütler, 2010). In other words, the amounts and spatial distribution of structural elements, such as deadwood, required at the landscape scale to allow for functional connectivity of taxa dependent on old forests have not received sufficient attention (Müller & Bütler, 2010; Percel, Laroche, & Bouget, 2019; Ranius & Fahrig, 2006). Increasing the knowledge of the minimum landscape‐scale requirements for biodiversity conservation through retention forestry would help to improve existing retention schemes (Mori et al., 2017).

Evidence‐based prescriptions for the amount and distribution of retention elements, however, will not guarantee the implementation of recommended measures into forestry practice. Successful biodiversity conservation in multi‐functional forests necessitates that conservation objectives are compatible with the aspirations of landowners and other goals of forest management (BMELV, 2011), and that potential trade‐offs are known and regulated by respective conservation policies. Conservation measures often represent a loss in income, cause issues of work‐safety, and result in higher management costs for forest owners (Rosenkranz, Seintsch, Wippel, & Dieter, 2014). Hence, the success of biodiversity conservation approaches such as retention forestry is greatly affected by the socio‐economic context, including local knowledge, traditions, motivations, and practices; yet, little is known about these human dimensions underlying the conservation of forest biodiversity in Central Europe and elsewhere (Bennett et al., 2017; Gorenflo & Brandon, 2006; Maier & Winkel, 2017; Rutte, 2011).

The inter‐ and transdisciplinary approach and the multi‐scaled design of the Research Training Group ConFoBi (Conservation of Forest Biodiversity in Multiple‐use Landscapes of Central Europe) explicitly address these two major gaps in forest biodiversity research, namely the influences of the landscape context and the relevance of the socio‐economic context for the effectiveness of retention measures to maintain biodiversity in multi‐functional forests of temperate Europe. To the best of our knowledge, such a broad integrative analysis of the ecological and social preconditions for forest biodiversity conservation has not yet been attempted in Central Europe.

In the following, we first present the rationale and lead questions underlying the ConFoBi research programme, then outline how these ideas were translated into a research design, describe how this design was implemented in the Black Forest, south‐west Germany, characterize the pool of 135 ConFoBi study plots and place the study system in a European context. We then briefly present specific projects, their methods and linkages. Finally, we discuss perspectives of ConFoBi for research and conservation, and encourage scientists from multiple disciplines to join the ConFoBi Research Training Group.

## 2 RATIONALE OF THE CONFOBI RESEARCH PROGRAMME

### 2.1 Guiding principles

The ultimate goal of ConFoBi is to provide an evidence‐based framework for socio‐political decision‐making, which rests on a comprehensive analysis of retention forestry and its consequences for and linkages to ecological and societal systems in the multiple‐use landscapes of temperate Europe. Key principles guiding ConFoBi's research toward this goal are (a) the application of a transdisciplinary approach, (b) the development of a consistently interdisciplinary research programme, and (c) the implementation of a real‐world representative study system spanning across multiple spatial scales, from plot (1 ha) to landscape.

#### 2.1.1 Transdisciplinarity

Biodiversity conservation often fails because of a lack of communication and understanding between researchers and practitioners (Mehring, Bernard, Hummel, Liehr, & Lux, 2017; Pregernig, 2014; Tinch et al., 2018). To overcome this deficiency, ConFoBi chose to apply a transdisciplinary approach to maintain a regular dialogue between science and practice. At the outset, we held a workshop with relevant stakeholders, which allowed us to orient research toward the knowledge gaps of forestry and conservation practice. Participants from different administrational levels within the State of Baden‐Württemberg, Germany, suggested that decision makers need (a) quantitative values for minimum amounts and distribution of retention elements required for forest biodiversity conservation at spatial scale extents from plots to landscapes and (b) knowledge related to local implementation practices of biodiversity conservation measures and instruments. Now, that ConFoBi is operational, a number of its research projects are designed and carried out in close cooperation with relevant decision‐making and managing bodies; especially the Ministry of Rural Affairs and Consumer Protection of Baden Württemberg (MLR) and the State Forest Service (ForstBW) are explicitly targeted at pertinent state policies, including the Forest Conservation Strategy (Waldnaturschutzstrategie; ForstBW, 2015), and the Old and Dead Wood Programme (AuT Programm; ForstBW, 2016).

#### 2.1.2 Interdisciplinarity

Successful biodiversity conservation in multi‐functional forests requires a solid ecological basis, but lastly depends on the compatibility of conservation measures with the human dimensions of forest management. In response to these requirements, ConFoBi adopted also an explicitly interdisciplinary approach, integrating multi‐scale ecological studies of forest biodiversity with social and economic studies of the preconditions and consequences of biodiversity conservation. To maximize interdisciplinary synergies, ConFoBi researchers of numerous disciplines work in the same set of study plots and surrounding landscapes and hence also in the same socio‐economic environment.

#### 2.1.3 A real‐world study system

Experiments have played an important role in research on the effectiveness of retention forestry (see Gustafsson et al., 2012 for an overview). Experimental results will generate valuable knowledge on the *potential* outcomes of retention approaches. However, whether these results are transferable to the conditions of real landscapes under different types of multi‐functional forest management remains questionable (Mori et al., 2017). In order to study retention effects in a setting representative of regular forestry practices, and to generate results directly relevant to forest owners and practitioners (see above, Section 2.1.1), ConFoBi is using plots in state‐owned forests available for wood supply, as a real‐world study system for its research programme. ConFoBi concentrates its research in a specific region, the Black Forest in southern Germany, with its specific forestry and biodiversity conservation structures and practices, to allow for transdisciplinarity. While the study system is regional, the findings are interpreted within a larger framework to achieve relevance for temperate forests with continuous‐cover forestry across Europe.

### 2.2 Research programme and lead questions

**Figure 1:**
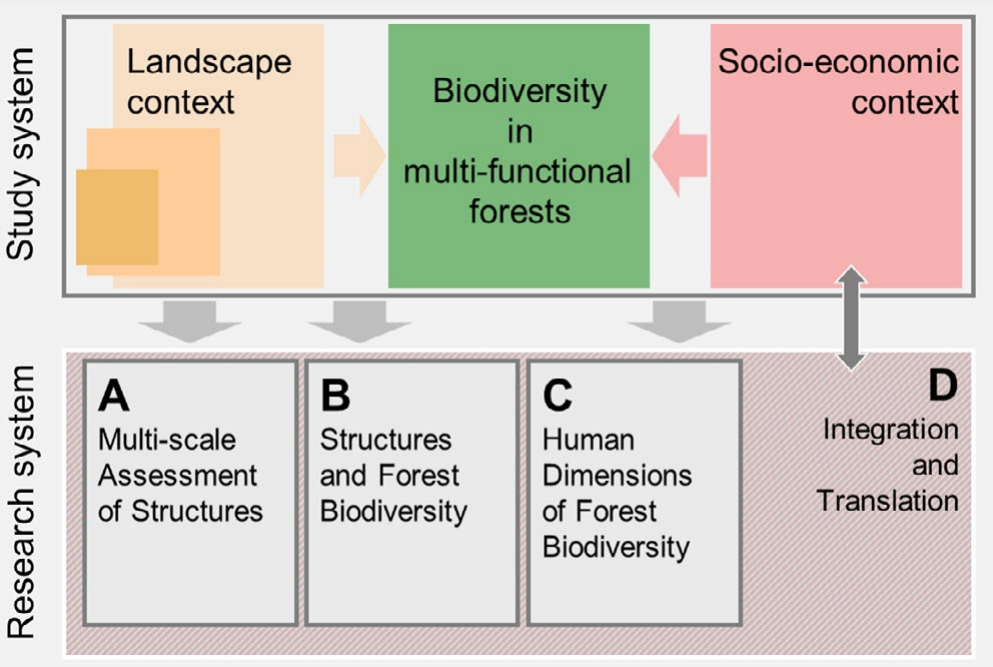
Illustration of the ConFoBi research concept. The study system (top) is represented (gray arrows) in a research system (bottom) consisting of four modules

ConFoBi assesses whether and how structural retention measures contribute to the conservation of forest biodiversity and analyses the potential of retention forestry to be adopted by landowners and be supported by stakeholders, using montane forests of the Black Forest, Germany, as a model system. ConFoBi will explicitly concentrate on the influences of the landscape context and the socio‐economic context on the effectiveness of retention measures to maintain biodiversity in uneven‐aged and selectively harvested continuous‐cover forests (Figure 1).

**Figure 2:**
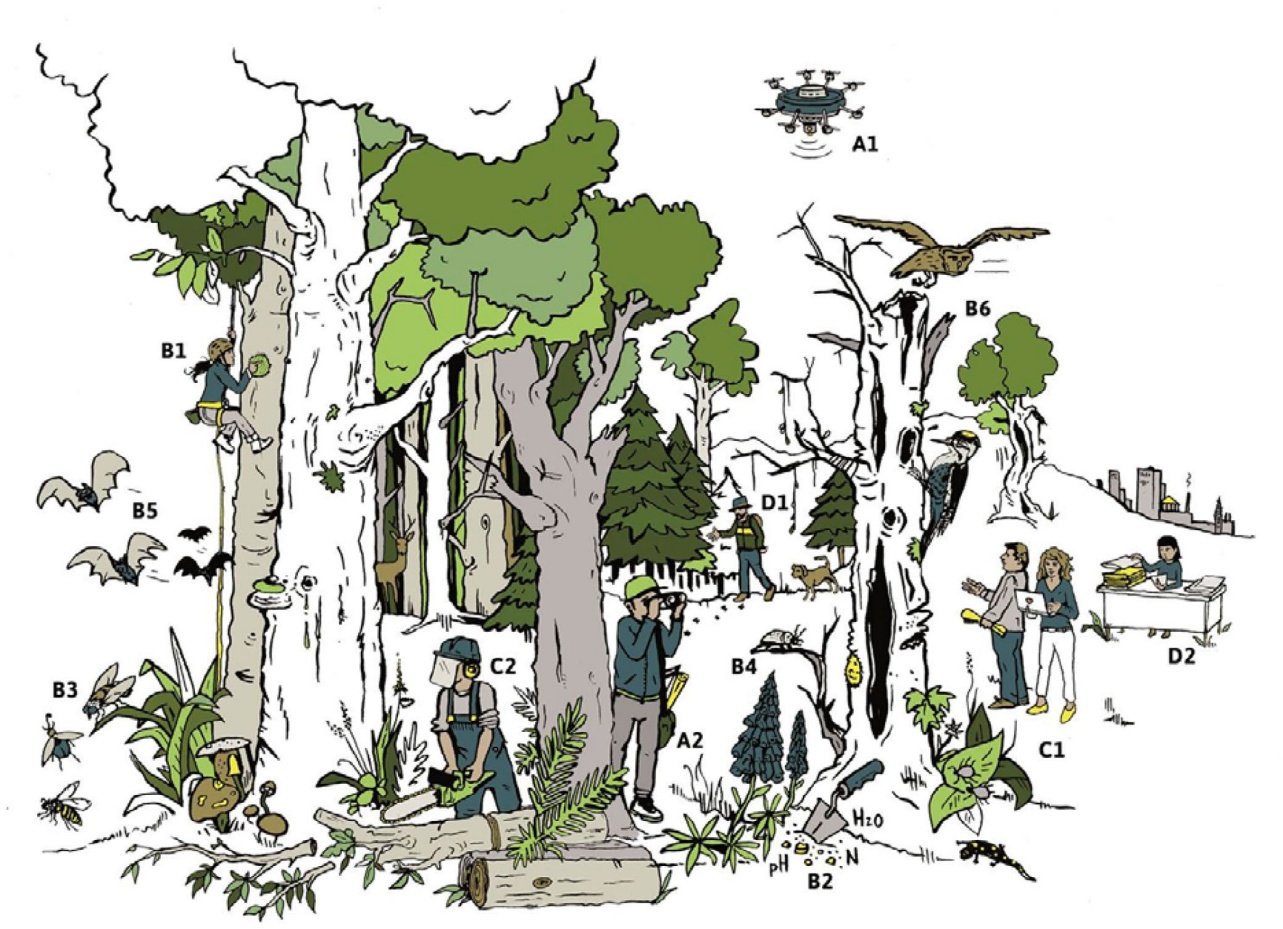
A cartoon of ConFoBi's interdisciplinary approach. All projects share the same study system with its 135 study plots but focus on different predictors, components, and drivers of forest biodiversity in a typical multiple‐use landscape of Central Europe. Letters and numerals indicate individual projects of Research Modules A–D (compare Figure1) (Illustration: Flimmern DC)

The structure of the collaborative research within ConFoBi consists of four modules A–D (Figures 1, 2 and 2) with several projects each. Module A provides tools for multi‐scale assessment of structures ranging from trees to landscapes. Module B studies a wide range of taxa (understory vegetation and epiphytes, invertebrates, mammals, birds) and relates biodiversity‐relevant metrics such as species occurrence, richness, and diversity to (a) plot‐scale (1 ha) forest structure, that is, abundance, quality, heterogeneity, and spatial distribution of structural elements within the forest and (b) to the landscape context, that is, abundance, quality, heterogeneity, and spatial distribution of forests in the surrounding landscape. Among structural elements, the focus is on habitat trees (large trees with hollows, cracks, crevices, crown deadwood, and other habitat features sensu, Bütler, Lachat, Larrieu, and Paillet, 2013) and deadwood, whereas site conditions and tree species compositions will be considered as covariates. Module C uses a subset of the ConFoBi plots selected along social gradients (e.g., ownership, protection status) to assess how forest practitioners in different settings perceive and practice biodiversity conservation, and will assess costs and benefits to model and optimize the economic efficiency of retention measures. Using a translational approach (Musacchio, 2009; Schlesinger, 2010), module D focuses on the interface between science and practice to assess how knowledge is generated and evidence is translated into practice, and to provide integration and communication between ConFoBi and forest and conservation managers and policy makers. In summary, ConFoBi:

- investigates the effects of forest structure including structural elements such as habitat trees and deadwood, and landscape context on multiple taxa including different trophic levels and functional groups (Modules A, B);
- analyses how forest biodiversity conservation is perceived and practiced, and what costs and benefits it creates (Module C); and
- identifies how biodiversity conservation can be effectively and efficiently integrated in multi‐functional forest management through a translational approach focusing on epistemologies (how knowledge is generated and transferred) and by developing evidence‐based guidelines for practitioners (Module D).

## 3 DESIGN AND IMPLEMENTATION OF THE CONFOBI STUDY SYSTEM

### 3.1 The Black Forest as a model system

ConFoBi has implemented its research programme in the southern Black Forest, Germany, as a model system for temperate forests. The Black Forest is a forest‐dominated low mountain range within a multiple‐use landscape typical of central Europe. It extends over an area of about 5,000 km^2^ and has a forest cover of 75% (365,000 ha). In the western and southern parts, the geology is dominated by granite and gneiss, whereas in the eastern and northern parts, sandstone prevails. The macroclimate is strongly influenced by the elevational gradient, ranging from 120 to 1,493 m a.s.l., with an average annual temperature between 4°C at the higher elevations and 10.4°C in the lowlands (Gauer & Aldinger, 2005). Norway spruce (*Picea abies* (L.) H. Karst.) is the most important tree species (42.8%), in particular in the northern and eastern Black Forest, whereas silver fir (*Abies alba* Mill.) (18.5%) and beech (*Fagus sylvatica* L.) (15.3%) maintain a higher share of the tree species composition in southern and western parts. In recent decades, the forests in the Black Forest have become older, more mixed and more structurally diverse (Kändler & Cullmann, 2016). In 2012, 71.5% of the forested area was covered by mixed stands, 29.5% was stocked with trees older than 100 years, two‐layered and multi‐storeyed forests made up 55% and 26%, respectively (Kändler & Cullmann, 2016). The average amount of deadwood (>10 cm diameter, incl. stumps >20 cm diameter) is 33.4 m^3^/ha, and there are on average five habitat trees per ha; the latter comprise 83% deciduous trees and more than two‐thirds of these trees have a diameter (dbh) >50 cm (Kändler & Cullmann, 2016).

The forested landscape is further characterized by a fine‐scale mosaic of ownerships, with some regional differences in the relative share of ownership types. In the Black Forest 39% of forests are privately owned, 27% are state owned, and the remaining 34% are owned by municipalities and public corporations (Kändler & Cullmann, 2016).

Forests in the region are managed using a variety of silvicultural systems. Owing to strict forest legislation, a prevailing paradigm of close‐to‐nature forest management, and the wide coverage of certifications systems (PEFC and FSC), clear‐cutting is basically not practiced in the Black Forest. Instead, regeneration methods such as shelterwood, group shelterwood (“Femelschlag”), strip cutting, and single‐tree selection (“Plenter” forest) are employed to create and maintain structurally diverse and species‐rich continuous‐cover forests (Bauhus & Pyttel, 2015), although the selection systems tend to disadvantage light demanding species such as pine and oak (Bauhus, Puettmann, & Kuehne, 2013).

In the State of Baden‐Württemberg, including the study area, a retention programme (“AuT‐Konzept” [translated as: old and dead‐wood programme]; ForstBW, 2016) stipulates the retention of one group of habitat trees (“Habitatbaumgruppe”) consisting of about 15 trees per 3 ha throughout state forests. Moreover, efforts have been taken to assist forest biodiversity on private property through contractual nature conservation, extension services, or subsidies.

### 3.2 Selection and establishment of study plots

The study design of ConFoBi is based on an “all‐measurements‐on‐all‐plots” approach with a common pool of 135 quadratic study plots (1 ha) distributed along two environmental gradients: (a) landscape‐scale forest connectivity (measured by the proportion of forest in the 25 km^2^ surrounding plot centers) and (b) retention‐related forest structure at the plot scale, that is, richness in habitat trees and deadwood per ha. For logistic and authorization reasons, all plots were selected in state forests and outside of wildlife protection areas with restricted access.

Plots were preselected on the basis of a set of criteria (Table 1) to reduce variation in confounding factors. Major criteria were topography (<35° slope, >500 m a.s.l.), stand age (>60 years), and the absence of waterbodies and human infrastructures. The remaining areas were classified into three forest‐connectivity classes (<50%, 50%–75%, >75% forest) based on the amount of forest in the surrounding landscape, as calculated within a circular moving window of 25 km^2^ based on a raster map of 25 × 25 m resolution.

**Table 1:** Criteria used to identify potentially suitable plots as well as geodata sources used for plot selection. After preselection based on the general criteria, potential plots were classified according the two design gradients, forest structure, and landscape pattern

Selection stage	Criterion	Feature/definition	Source
Preselection	Forest ownership	State owned	Forest inventory data, Geodata service of the Forest administration of Baden‐Württemberg (FGeo)
	Region	southern Black Forest, Baar‐Wutach	Ecoregions according to Aldinger et al. (1998), Forest Research Institute of Baden‐Württemberg
	Elevation	≥500 m a.s.l.	Digital elevation model (DEM), aggregated to 25 × 25 m resolution; State Agency of spatial information and rural development of Baden‐Württemberg (LGL), https://www.lgl-bw.de/lgl-internet/opencms/de/05_Geoinformation/Geotopographie/Digitale_Gelaendemodelle/ (30 October 2015)
	Steepness of slope	≤35°	DEM, State agency of spatial information and rural development of Baden‐Württemberg (LGL), https://www.lgl-bw.de/lgl-internet/opencms/de/05_Geoinformation/Geotopographie/Digitale_Gelaendemodelle/ (30 October 2015)
	Stand age	≥60 years	Forest inventory data, Geodata Service of the forest administration of Baden‐Württemberg (FGeo)
	Distance between plot centers	>750 m	GIS
	Infrastructure (buildings, roads)	(excluded)	ATKIS^®^, State Agency of spatial information and rural development of Baden‐Württemberg (LGL); Amtliches Topographisch‐Kartographisches Informationssystem. http://www.lgl-bw.de/lgl-internet/opencms/de/05_Geoinformation/AAA/ATKIS/ (30 October 2015)
	Waterbodies	(excluded)	ATKIS^®^, State agency of spatial information and rural development of Baden‐Württemberg (LGL)—Amtliches Topographisch‐Kartographisches Informationssystem. http://www.lgl-bw.de/lgl-internet/opencms/de/05_Geoinformation/AAA/ATKIS/ (30 October 2015)
	Restricted species protection areas	(excluded)	Geodata service of the Forest Research Institute FVA
Landscape‐scale forest‐connectivity gradient	Forest within surrounding 25km^2^	3 classes: <50%, 50%–75%, >75%	ATKIS^®^, State agency of spatial information and rural development of Baden‐Württemberg (LGL)—Amtliches Topographisch‐Kartographisches Informationssystem. http://www.lgl-bw.de/lgl-internet/opencms/de/05_Geoinformation/AAA/ATKIS/ (30 October 2015)
Forest structure gradient	*N* standing dead trees within 1‐ha plot	3 classes: 0, 1–9, >10	Stereo color‐infrared aerial images of 2015, State agency of spatial information and rural development of Baden‐Württemberg (LGL)

To determine the structural gradient, using designated habitat trees of the AuT‐Programme was not an option. At the time of plot selection, in 2016, AuT was still in its initial implementation stage, habitat trees had been selected for only minor parts of the study, and designated habitat trees were not (yet) older than the surrounding stands. Instead other proxies for forest structures that are expected to be enhanced by retention forestry, that is, old or dead trees rich in microhabitats, were used for plot selection. We screened the study area for 1 ha‐plots with low (0), medium (1–9), and high (≥10) numbers of standing dead trees. Trees were visually assessed using stereo aerial color‐infrared imagery, as provided by the state agency of spatial information and rural development of Baden‐Württemberg (LGL), together with a stereo viewer. Fifteen plots from each category (low, medium, and high structure) were then randomly selected for each of the forest‐connectivity classes, ensuring that the centers of two neighboring plots were >750 m apart, resulting in 135 plots arranged in a stratified design with 3 × 3 = 9 categories.

**Figure 3:**
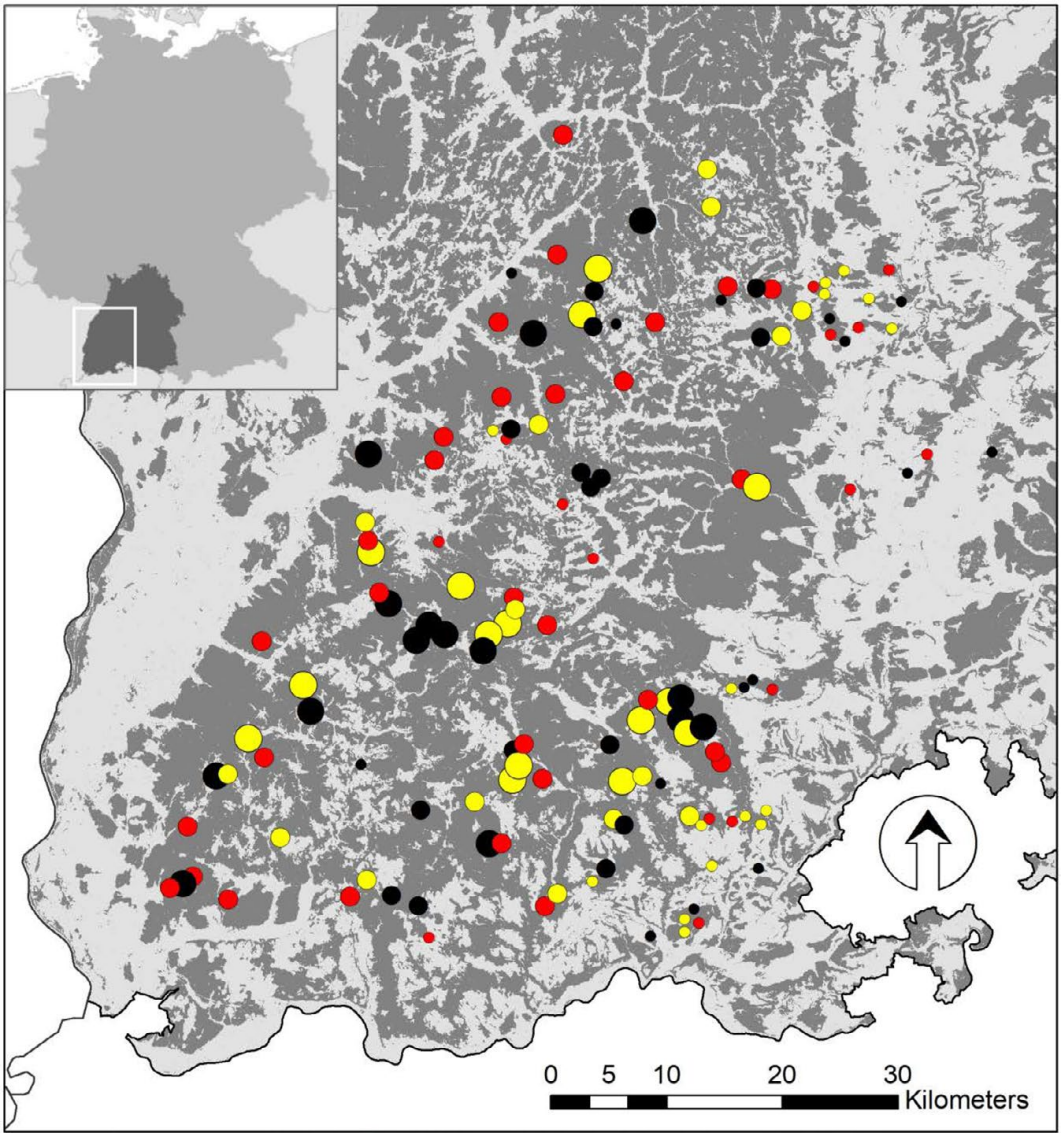
Location of the 135 ConFoBi study plots in the Black Forest (main map; light gray: open land; dark gray: forest), and in Germany and the State of Baden‐Württemberg (insert). All plots are 1 ha in size and >750 m apart. Point size indicates three levels of landscape‐scale forest connectivity (small <50%, medium 50%–75%, large >75% forest cover) in the 25 km^2^ surrounding a plot. Point color indicates three levels of plot‐scale forest structure (red: 0, yellow 1–9, black ≥20 standing dead trees per ha). Plots richest in structure (≥20 standing dead trees) include stands >200 years of age and plots in strict forest reserves, where harvesting has been excluded

These 135 candidate plots were cross‐checked with the local forest managers for the possibility to largely exclude forestry operations (e.g., harvesting and road construction) on these plots until the end of the anticipated maximum ConFoBi funding period (2016–2025). Individual plots, for which operations were already scheduled, were randomly replaced by plots of the same category. The final plots were confirmed in agreement with the individual State Forest Offices in charge of the respective forest stands. Of the 135 final ConFoBi plots, 115 are located in multi‐functional forests regularly managed by the State Forest Service; another 20 plots (all belonging to the category richest in deadwood) are in strict forest reserves (without any harvesting of timber). Plots are distributed as shown in Figure 3.

This 3 × 3 (=9 plot categories) ordinal design was used for securing an even distribution of plots along the two design gradients; subsequently, however, plot‐scale forest structure and landscape‐scale forest connectivity were measured using numerous continuous metric variables (see Figures 5 and 6, Appendix 1). The sample size of 135 plots was chosen to compromise between statistical power and logistic limitations.

**Figure 4:**
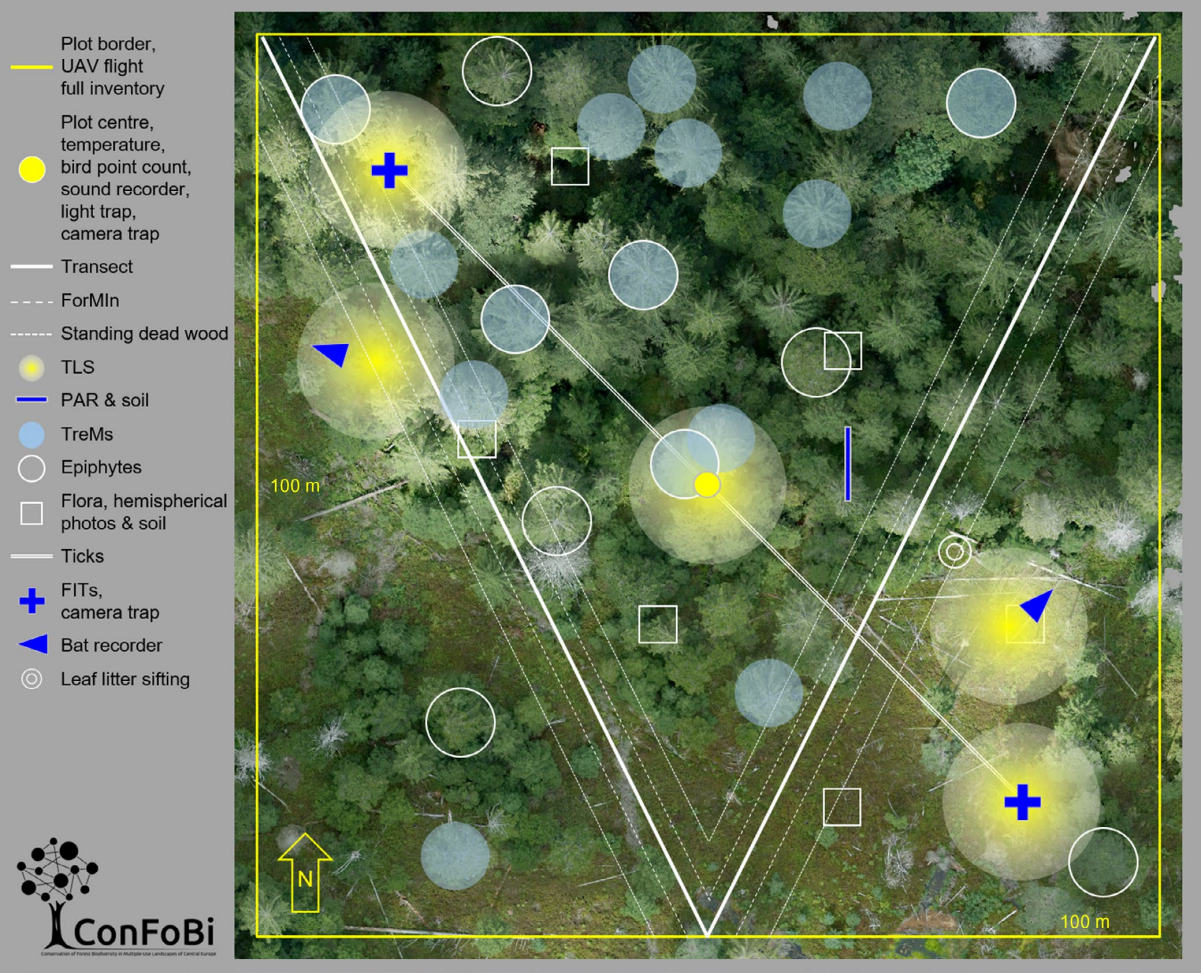
Schematic overview of the sampling design on the ConFoBi study plots. All plots are 1 ha in size and north south aligned. Wooden poles mark the plot center as well as all four corners and all four sides. The center point is permanently marked with a white plastic reference point and a strong magnet on ground level. Trees along the borders and in the corners of the plot are also marked with long lasting light blue color. The following measurements are collected on all 135 study plots, except for a few additional measurements, for example, for pilot studies, that are taken on subsets of plots (*n* plots in brackets) only: Flights with unmanned aerial vehicles covering the whole plot, within the whole plot full inventory of all trees with a DBH above 7 cm as well as full presence list of all herbaceous plants; at the center point temperature, bird counts, automatic acoustic recorders for soundscapes, light traps for moths (*n* = 28); automatic camera traps for large mammals at the center point and the locations of the Flight Interception Trap (FIT); a V‐transect is aligned from the north‐western corner via the central point of the southern border to the north‐eastern corner, data for the ForMIn (Forest management Index) within a 4m wide strip and data on standing deadwood within a 10 m wide strip are collected; terrestrial laser scans (TLS) on five locations (plotcenter, two bat recording, two insect collecting sites); light measurements (photosynthetic active radiation = PAR) along a transect subdivided into twelve subplots of 40 cm × 40 cm plus one soil sample from the middle of the transect, the transect was placed north south in the grid cell of 10 m × 10 m with the highest variability of crown height of each study plot; tree microhabitats (TreMs) on the fifteen trees with the largest crown identified from aerial images; epiphytes on the five trees with the largest crown identified from aerial images and on five trees of the most common species and of average DBH of each plot; ticks are collected with a 1 m × 1 m flag along a 100 m transect aligned north‐west to south‐east through the center point in four 25 m steps (*n* = 34); six floral subplots of 5 m × 5 m which detail species list plus cover, in addition one soil sample and one hemispherical photograph were taken at the center of each subplot; FIT in the north‐western and south‐eastern area of the plot; automatic acoustic bat recorders placed in one area of the plot with high structure and one area with low structure; sifting leaf litter for weevils, centipedes, and millipedes along deadwood next to beech trees (*n* = 43)

Plots were established in the field by marking the center with a metal rod and a magnet at ground level. Afterward the borders were identified with a differential GPS and marked. A schematic overview of sampling design on the plots is shown in Figure 4.

All plots were inventoried by measuring the DBH of every tree (above 7 cm DBH) and the amounts of lying and standing deadwood. Because habitat trees are an important structural element in retention approaches, for each study plot we mapped the 15 largest trees (measured by crown area from aerial images) and quantified in great detail their existing microhabitats (such as hollows, cracks, and crevices; Larrieu et al., 2017). Abiotic measurements in the plots include air temperature at 150 cm height at 1‐hr intervals in the center of every plot and quantification of light levels at the understorey, as well as soil nutrients. Additional abiotic data (e.g., precipitation) are derived from existing sources on a landscape scale (atlas data). Landscape patterns have been described using a range of metrics (measured around plot centers), including amount of forest cover, land cover composition, and edge density (Appendix 1).

### 3.3 The study system in a European context

The ConFoBi study system is embedded in a typical multiple‐use landscape of Central Europe; its study plots are part of a forest landscape with a variety of silvicultural systems and ownership types. Yet, the selection of study plots ensured that a wide range of the conditions found in European montane forests are represented; this in particular holds for the two design gradients, amount of deadwood at the plot scale, and amount of forest at the landscape scale.

**Figure 5:**
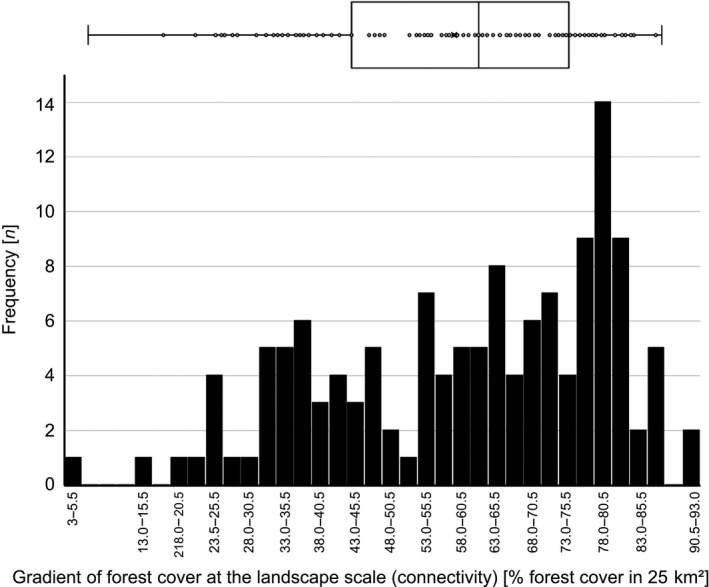
Gradient of forest cover at the landscape scale (connectivity). Boxplot and frequency distribution of the 135 study plots by forest cover in 25 km^2^ surrounding plotcenter

**Figure 6:**
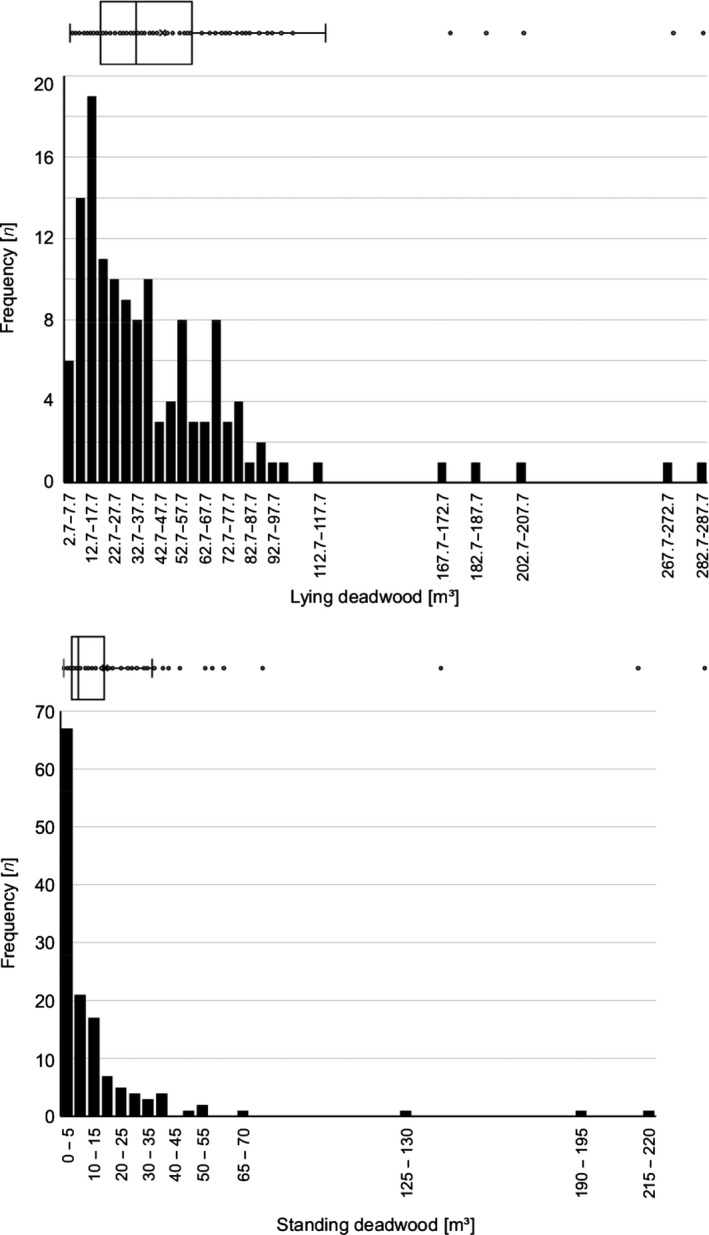
Gradients of forest structure at the plot scale. Boxplots and frequency distributions of the amount of lying (top) and standing (bottom) deadwood on the 135 study plots (1 ha)

The distribution of the 135 study plots along these two design gradients is shown in Figures 5 and 6. The landscape gradient ranges from a forest matrix with high connectivity, to highly fragmented forests with an open matrix (Figure 5). Mean volumes of standing and lying deadwood amount for 14.0 and 43.6 m^3^/ha, respectively (Figure 6). The plots richest in deadwood with close to 500 m^3^/ha are located in strict forest reserves. According to studies in forest reserves of temperate Europe, natural amounts of >200 m^3^/ha of standing and downed deadwood can be expected in montane beech‐fir forests (Bujoczek, Szewczyk, & Bujoczek, 2018). Temperate forests used for timber production typically are poor in deadwood (Vítková et al., 2018); on average, the volume of total deadwood is around 11.5 m^3^/ha in the forests of European countries, with standing deadwood making up for about one third, and lying deadwood for two‐thirds of the total volume (Forests Europe, 2015). For Germany, 4.7 and 15.9 m^3^/ha standing and lying deadwood, respectively, are reported (Forests Europe, 2015); for the State of Baden‐Württemberg, total deadwood amounts average 28.8 m^3^/ha, about half of which (14.1 m^3^/ha) is standing deadwood (Kändler & Cullmann, 2014); slightly higher amounts are reported for the Black Forest (total deadwood 33.4, lying 17.2 m^3^/ha) (Kändler & Cullmann, 2016). Thus, the ConFoBi study plots represent the full gradient of the deadwood amounts reported from managed as well as protected mixed montane forests in Central Europe, spanning from close to zero to volumes typical of natural stands. Further variables characterizing the ConFoBi study system at plot and landscape scales are presented in Appendix 1.

## 4 PROJECTS, METHODS, AND LINKAGES

Currently, there are 14 different ConFoBi projects, focussing on the topics and methods sketched in Box 1. Module A projects provide structural data at plot and landscape levels, which form a basis for analyses of biodiversity responses of various taxa to retention measures (B‐projects), and provide input for the study of economic implications of biodiversity‐oriented forest management, and biodiversity knowledge and conservation practices of forestry practitioners (C‐projects). All projects with an ecological or economic focus work on all 135 plots (Figures 1, 2 and 2). The remaining social sciences projects do not strictly work on the plots only, because the relevant unit of analysis is not a spatially delimited area, but rather, for example, a specific type of organization (like a forest enterprise, a forest owner association, a forestry training center or a conservation agency) in which biodiversity‐relevant decisions are being made. Since in the landscapes surrounding the 135 plots all types of land tenure and forest ownership (state, communal, and private) are represented, the social sciences projects are tightly linked to and integrated into the ConFoBi study system.

Additional projects with complementary research questions are under development; for example, recently initiated (pilot) studies address effects of forest structure on the abundance of ticks and transmittable diseases, moths, leaf litter‐inhabiting organisms, fungi, and salamanders.

Box 1 **A Structures**

**A1 Remote sensing based methods for the assessment of forest structures**

The project develops remote‐sensing methods to assess abundance, heterogeneity, and spatial distribution of structural elements, as predictors of biodiversity across multiple temporal and spatial scales. Advanced remote‐sensing techniques, such as LiDAR and digital stereophotogrammetry, in conjunction with platforms such as Unmanned Aerial Vehicles and terrestrial laser are used and new algorithms for extraction and unification of data are developed to provide structural information at scales appropriate for other ConFoBi projects.

**A2 Retention of structural elements in selectively used forests**

Live or dead standing trees that provide tree‐related microhabitats (TreMs) such as cavities, large dead branches, loose bark, epiphytes, bracket fungi, cracks, or trunk rot are defined as habitat trees. In natural forests habitat trees and TreMs may be highly clumped and variable, which may affect structure and viability of communities and meta‐communities of species reliant on microhabitats. A2 analyses and predicts TreMs on potential habitat trees, and assesses factors relevant for habitat tree selection, such as distribution, quality and longevity of habitat trees and TreMs, forest management intensity, and landscape context.

**B Biodiversity components**

**B1 Epiphyte and microhabitat diversity and function on habitat trees**

The project quantifies the role of habitat trees for conserving forest epiphytes, and assesses how habitat trees differ in their epiphytic lichen and bryophyte diversity from trunk up to the crown and in variation of the species identity of the habitat tree, the main tree species of the stand, the diameter and microhabitat characteristics of the habitat tree, and landscape‐scale forest connectivity.

**B2 Mechanisms of vegetation change and diversity in retention forestry**

The project addresses the role of habitat trees and deadwood, and their landscape context, for plant diversity by disentangling various effects of abiotic changes on plant performance. B2 determines the composition and spatial distribution of understorey higher plants and ground‐dwelling mosses, to assess how heterogeneity in forest structure influences resource heterogeneity and availability of light for understorey vegetation, and hence species diversity and composition, and to quantify the role of forest structure and resource heterogeneity on trait distributions and functional diversity of the understorey.

**B3 Diversity and functions of plant‐insect interactions along a retention gradient**

The project addresses the overall hypothesis that stand‐scale retention measures influence the diversity and trophic interactions of hymenoptera and other arthropods, which are mediated by forest composition and configuration. B3 analyses the relationship between components of insect diversity and environmental variables related to retention (e.g., deadwood, microhabitats). To quantify trophic interactions and food webs plant galls with their gall‐forming communities and cavity‐nesting Hymenoptera are studied.

**B4 Functional connectivity among saproxylic beetles in dead‐wood patches**

The project investigates species and genetic diversity of saproxylic beetles and hymenoptera, and assesses gene flow of dead‐wood specialists with different dispersal ability as a function of abundance, distribution and isolation of deadwood across the landscape. Species are sampled at dead‐wood patches of different size and distance by means of trapping and metabarcoding. Genetic diversity is quantified using metabarcoding for total diversity and species‐specific markers (RADSeq) for genetic distance. Finally, B4 infers thresholds for functional connectivity among dead‐wood patches.

**B5 Landscape‐moderated use of forest structures by bats**

The project assesses linkages between multi‐scale forest structures and bat activity, richness, and diversity, as well as the functional importance of structures, specifically for foraging and commuting. Automated acoustic recorders are deployed to detect bat species(‐groups) and to quantify their activity. Presence, richness, diversity, and activity of bats are then related to measures of forest structure, including LiDAR‐information capturing the 3D‐characteristics of subcanopy space, and landscape‐scale connectivity metrics. Availability of food will be assessed (with B3, B4, and B6) to address inter‐trophic relationships that may explain the observed results.

**B6 Multi‐scale assessment of bird‐forest relationships**

The project quantifies occurrence and abundance of bird species based on repeated aural‐visual point counts in order to assess multi‐scale structural predictors of avian diversity. B6 first focusses on linkages between retention elements, landscape patterns, and birds; thereafter, on this basis, B6 assesses inter‐trophic relationships of birds that may explain the patterns observed. Assessment of food availability (with B3–B5) will allow elucidating functional relationships.

**B7 Soundscapes**

This project examines how acoustic diversity can be quantified to reflect avian diversity, and how these metrics can be combined with other variables to describe a forests potential for biodiversity. To test the hypothesis that acoustic diversity of avian vocalizations varies in response to multi‐scale forest structures B7 collects audio files and analyses acoustic indices to identify and track phenological changes in avian communities. These indices are validated against observed species richness and abundance (B6), then related to additional taxonomic groups (B3–B5) and metrics of forest structure (A1, A2, B2) at the plot and landscape level to model optimal combinations of indices and quantitative benchmarks to predict biodiversity.

**B8 Ungulate‐forest relationships**

The project focusses on the relationship between roe deer *Capreolus capreolus* and forest structure, addressing both bottom‐up and top‐down effects. Local abundance and habitat selection of roe deer, quantified through camera trapping and pellet counts, are related to biomass and composition of understorey vegetation, and to forest structure and landscape pattern. Effects of deer on plant community composition and understory species richness are addressed through browsing surveys and germination experiments based on deer feces. Finally, co‐occurrence and direct and indirect biotic interactions among roe deer and other taxa (with B1‐6) are assessed.

**B9 Effects of forest structures on fungi**

The project quantifies the impact of multi‐scale forest structure and fragmentation on the diversity of fungi with focus on wood‐inhabiting species and interactions between fungi and other organismic groups. It combines standard monitoring techniques with genetic analyses of selected species to determine genetic differentiation among local populations along gradients of forest fragmentation. Taxonomic, functional and genetic diversity measures of fungi are related to various forest structural and landscape metrics, as well as to diversity and composition of plants and animals (B1‐8).

**C Human dimensions**

**C1 Economic valuation of biodiversity‐oriented forest management strategies**

The project hypothesizes that orienting forest management toward biodiversity has economic implications that can be quantified on different spatial scales. First, the value of biodiversity is expressed as an opportunity cost within a forward‐looking simulation‐optimization approach; thereafter, C1 quantifies social benefits of biodiversity, investigating optimal retention levels and spatial allocation of retention under multiple uncertainties including climate change. Finally, C1 aims to propose cost‐effective measures to promote biodiversity. For this, C1 collaborates with the A and B projects for structural and biodiversity data, and with C2 and D1 to find out which role economic parameters play in decision‐making related to biodiversity.

**C2 Local biodiversity knowledge and forest conservation practices**

This project connects three distinct fields of research. It conceptually links socio‐psychological findings of research on forest owners, their attitudes, practices and institutional integration and the work on traditional ecological knowledge and practices in conservation to the literature on forest and biodiversity conservation discourses and professional forest management paradigms among practitioners and policy makers. These questions have never systematically been related to each other in an empirical study. After a systematic review, exploratory interviews, and document analysis, qualitative interviews are combined with participatory observation. Finally, a representative mail survey will quantify the derived patterns.

**D Science‐practice interface**

**D1 Professional epistemologies and integration of biodiversity‐related knowledge into socio‐political decision‐making**

The project assesses under which conditions specific stocks of biodiversity‐related knowledge are taken up in different decision‐making contexts. D1 is built on the hypothesis that both within biodiversity science and biodiversity policy and management there are distinguishable professional epistemologies that have an impact on problem definition, agenda setting as well as the formulation and implementation of problem‐solving strategies. The project uses a qualitative‐interpretative approach to investigate the specific “thought styles” of ConFoBi‐relevant scientific disciplines as well as those found in practical decision‐making contexts. Major approaches are document analysis, expert interviews, and participatory observations.

**D2 Evidence‐based biodiversity management of forests**

This project explores the scientific basis of management principles for conservation in forests. One challenge is the plethora of studies of different design and quality, sometimes yielding equivocal conclusions about causal links between management and biodiversity. Experts integrate the information available to them into a belief, which may or may not be a reasonable summary of the state of knowledge. D2 aims to critically evaluate the foundation of such models, both mathematical and mental, and juxtapose them with scientific evidence in the literature. D2 compares three representations of understanding: general causal knowledge with high levels of published evidence; specific causal assumptions represented in mathematical and computer models with biodiversity as a state variable; and intuitive causal belief of biodiversity‐generating processes in scientists and forest managers.

## 5 PERSPECTIVES FOR RESEARCH AND CONSERVATION

### 5.1 Complementing other research efforts

To our knowledge, ConFoBi is the first research programme that investigates how retention of forest structures affects forest biodiversity in uneven‐aged and selectively harvested continuous‐cover forests of temperate Europe (Gustafsson et al., 2019). With its explicit focus on retention practices and by integrating the landscape and socio‐economic contexts of forest biodiversity, ConFoBi complements other research efforts on forest biodiversity. Synergies mayarise with the “Biodiversity Exploratories” (Fischer et al., 2010), “FunDivEUROPE” (Baeten et al., 2013) and its follow‐up process “SoilForEUROPE” (http://websie.cefe.cnrs.fr/soilforeurope/), as well as small FOREST (Valdés et al., 2015). Other related projects include “L57” (Management of species diversity in integrative forestry, http://www.waldbau.wzw.tum.de/index.php?xml:id=155), “BioHolz” (Biodiversity and Ecosystem Services of Forests, https://www.bioholz-projekt.de) and “RTG 2300: Enrichment of European beech forests with conifers” (https://www.uni-goettingen.de/en/574316.html). These projects also work on biodiversity in forests, but do not explicitly address effects of retention measures on biodiversity. Finally, ConFoBi is collaborating with the European Forest Institute's (EFI) INFORMAR project focussing on socio‐economic driving factors that determine the scope for implementing integrated forest management approaches across Europe, and with regard to science‐policy‐practice interface activities to strengthen the practice and policy impact of its work internationally.

### 5.2 Knowledge and expertise for integrated forest management

ConFoBi's research results will lead to quantitative target values describing the optimal amount, quality, and distribution of retention sites for different species and taxonomic groups as well as to recommendations how to efficiently integrate these targets into multi‐functional forest management. ConFoBi will provide an extensive case example for both reseach and management of forest biodiversity across the multiple‐use landscapes of the temperate zone.

In its first stage, ConFoBi focusses on describing patterns of biodiversity in relation to forest structures at spatial scales from plot to landscape. In later stages, the focus will shift from biodiversity patterns to functional relationships (e.g., between forest structures and organisms; among taxa and across trophic levels) and processes, which shape the observed patterns. To understand the socio‐economic context of biodiversity conservation in managed forests, ConFoBi will move from describing general knowledge structures to actions and impacts, to assess how practitioners integrate conservation in forest management. Finally, in an interdisciplinary synthesis, ConFoBi will quantify retention targets, and in particular the amounts and distribution of habitat trees and deadwood, required across the landscape for effective biodiversity conservation, identify the trade‐offs between forestry and biodiversity conservation, and will elaborate the socio‐economic prerequisites for their implementation by forest owners and managers. Wider applicability of the results will be ensured in cooperation with research groups elsewhere, for example, in the newly established COST Action BOTTOMS‐UP (https://www.cost.eu/actions/CA18207/#tabs%7CName:overview).

ConFoBi emphasizes the ecological foundations and socio‐economic frameworks of retention approaches in continuous‐cover forestry (Gustafsson et al., 2019). Preliminary results indicate positive overall effects of retention of structural elements for forest biodiversity but also high variability among taxa and landscape settings; further, our findings suggest high potential for optimizing the integration of retention into management practice. The first ConFoBi publications described technical and methodological advances for quantifying forest structures; for example, we found a high potential for optimizing the quality in reconstruction of 3D forest models from aerial images based on drones (Frey, Kovach, Stemmler, & Koch, 2018), showed how the abundance and diversity of tree‐related microhabitats can be predicted with readily available forest attributes (Asbeck, Pyttel, Frey, & Bauhus, 2019), recorded a lichen species new for Germany (Wirth, Tønsberg, Reif, & Stevenson, 2018), optimized the trap design to capture flying arthropods (Knuff, Winiger, Klein, Segelbacher, & Staab, 2019), and showed that the occurrence of specialist herbivore communities might be best explained by plant species composition rather than the abiotic environment (Knuff, Staab, Frey, Helbach, & Klein, 2019). Based on meta‐analyses, ConFoBi researchers confirmed that crown‐damaged trees improve nesting opportunities for cavity‐nesting birds (Gutzat & Dormann, 2018) and that woodpeckers select cavitiesby relative rather than absolute tree size (Basile, Mikusinski, & Storch, 2020), but found that bird guilds are affected differently by forestry measures including retention, according to their life history, biome, and forest type (Basile, Mikusinski, & Storch, 2019). A joint study by social and remote‐sensing scientists of ConFoBi found expert ratings of forest structure, despite large individual bias, were on average significantly related to technical structural complexity indices based on terrestrial laser scanning (Frey, Joa, Schraml, & Koch, 2019), and a review concluded that local ecological knowledge holds significant promise for integrating conservation objectives into forest management under changing environmental conditions (Joa, Winkel, & Primmer, 2018; Joa & Schraml, 2018). Analyses of the opportunity costs arising from retention forestry suggest that conservation practices, such as habitat networks of deadwood islands, will only marginally impact profitability when conservation and production goals are balanced through suitable planning tools (Augustynczik, Yousefpour, Rodriguez, & Hanewinkel, 2018). Interdisciplinary modeling suggested that integration of uncertainty into conservation planning may reduce the trade‐off between production and conservation objectives in forest landscapes (Augustynczik et al., 2017; Augustynczik, Yousefpour, & Hanewinkel, 2018), and a diversification of forest management regimes is recommended for securing various model taxa, including saproxylic beetles (Augustynczik, Yousefpour, et al., 2018), as well as tree microhabitats and birds under climate change (Augustynczik, Asbeck, et al., 2019; Augustynczik, Yousefpour, et al., 2018). Overall, ConFoBi's analyses suggest that current forest management for biodiversity is inefficient under climate change (Augustynczik et al., 2020; Augustynczik, Yousefpour, & Hanewinkel, 2019).

ConFoBi's results and recommendations will be translated for practice and political decision makers using the established science‐practice‐policy communication pathways of the collaborating partners Forest Research Institute of Baden Württemberg (FVA) and European Forest Institute (EFI). National and State Agencies directly concerned with forests and conservation and other national‐level and EU‐level decision makers are involved in a transdisciplinary dialogue throughout ConFoBi. ConFoBi will identify the ecological as well as the socio‐economic prerequisites for effective implementation of retention measures for biodiversity by forest owners and managers. ConFoBi's results will be seminal for integrating biodiversity conservation into forest management by providing an interdisciplinary evidence base for optimizing the effectiveness of retention approaches.

### 5.3 Complementing ConFoBi: an invitation

While ConFoBi is primarily a Research Training Group for doctoral students, we have also set it up to provide a research platform that can be extended in scope, spatial, and temporal scales, as well as interdisciplinarity. Whereas the first set of studies primarily addresses disciplinary questions, later studies will focus on synergies across projects and disciplines. Finally, ConFoBi will synthesize and validate its results on the relationships between forest biodiversity, retention measures, and their landscape and socio‐economic contexts, using a range of approaches from empirical studies to scenario modeling. ConFoBi is open to complementary projects. Scientists from all career levels—doctoral students to senior researchers—and from all disciplines are welcome to propose complementary research ideas within the framework of ConFoBi; PhD students and PostDocs are particularly invited; inter‐ and transdisciplinary studies are strongly encouraged. Further information on ConFoBi as well as information on study sites and datasets generated within ConFoBi will be made available here: http://confobi.uni-freiburg.de/en and can be requested from the corresponding author.

## CONFLICT OF INTEREST

Authors declare no conflict of interest.

## AUTHOR CONTRIBUTIONS

All authors (IS, JP, TA, MB, JB, VB, CFD, JF, SG, MH, BK, A‐MK, TK, MP, PP, AR, MS‐L, GS, US, MS, GW, and RY) have contributed to the development of the research programme and study design of ConFoBi, and/or the selection, establishment, and inventory of the study plots. The lead author (IS) has written the manuscript and is the spokesperson of ConFoBi; the second author (JP) collated the plot data, all remaining authors are listed in alphabetical order. All authors contributed to drafts and gave final approval for publication.

## Data Availability

Data sharing is not applicable to this article as no new data were analyzed in this article. The datasets used to illustrate the distribution of the study plots along variables of forest structure and landscape connectivity (Figures 5 and 6) are available from the corresponding author on reasonable request.

## APPENDIX 1

Examples of measures of topography, vegetation, and landscape pattern for the 135 ConFoBi study plots. Topography and forest characteristics are mean values and total counts, respectively, referring to 1 ha; landscape metrics were calculated for various scales (see Schindler, Wehrden, Poirazidis, Wrbka, & Kati, 2013; respective moving window sizes are given in brackets). Definitions of landscape metrics according to McGarigal (2015). Note that Landsat forest classes (conifer, broad‐leaved, and mixed) were merged into class “forest,” remaining classes to “nonforest.”

**Table:**

Measure	Unit	Definition		Source	Range (min–max)	Mean	*SD*	Reference
Topography								
Elevation	m a.s.l.		Mean value derived from 1 m digital terrain model	LGL (2005)	443–1334	822	±182	
Slope	Degree		Mean value derived from 1 m digital terrain model	LGL (2005)	1–34	15	±9	
Aspect	Degree		Mean value derived from 1 m digital terrain model	LGL (2005)	3–360	172	±109	
TRI (Terrain ruggedness index)	m		Mean value of the mean difference between a central pixel and its surrounding cells derived (moving window) from 40 cm GSD DSM (Ground Sampling Digital Surface Model) generated from 20 cm aerial images using SfM (Structure from Motion)	ConFoBi data	0.25–0.97	0.56	±0.16	Wilson, O'Connell, Brown, Guinan, and Grehan (2007)
Vegetation								
No of trees	*N*		Inventory of all trees inside 1 ha plot with DBH >7 cm	ConFoBi data	98–1212	425	±205	
Tree species	Species		Inventory of all trees inside 1 ha plot with DBH >7 cm	ConFoBi data				
DBH	mm		Inventory of all trees inside 1 ha plot with DBH >7 cm	ConFoBi data	70–1268	271.0	±166.0	
Basal area living trees	m^2^		Inventory of all trees inside 1 ha plot with DBH >7 cm	ConFoBi data	9.4–73.1	34.1	±9.9	
Tree height	m		Mean value derived from subtraction of digital terrain (DTM) model from calibrated surface heights from UAV‐SfM (Unmanned aerial vehicle‐Structure from Motion) flights	DTM: LGL 2005; UAV: ConFoBi data	8.6–40.6	24.1	±5.9	Frey et al. (2018)
Standing deadwood	*N*		Calculated from plot inventory	ConFoBi data	0–394	33.4	±53.6	
Basal area standing deadwood	m^2^		Mean value derived from plot inventory (BA = 0.00007854 × DBH^2^)	ConFoBi data	0–51.2	2.2	±5	
Standing dead‐wood volume	m^3^		Calculated from plot inventory (*V* = Basal area × height × 0.5 (form factor))	ConFoBi data	0–2163	140	±282	
Lying dead‐wood volume	m^3^		Calculated from described *V* transect (*V* = (k/*L*)∑*d* ^2^; k = constant = 1.234 (see Van Wagner, 1982), *L* = length of transect, *d* = DBH)	ConFoBi data	2.7–282.9	43.6	±43.7	Van Wagner (1982), Kahl and Bauhus (2014)
NDVI			Normalized Difference Vegetation Index; mean value derived from Sentinel 2 data	ESA (2018)	0.61–0.82	0.72	±0.036	Rouse, Haas, Schell, and Deering (1973)
Landscape								
Heterogeneity as proportion of stands	%	Derived from stand based local forest inventory of Baden‐Württemberg		FoGIS10/InFoGIS (MLR) (2018)	0.001–100	54.0	±39.5	
Distance from plot center to nearest forest edge	m	Value derived from OpenStreetmap‐Data		OpenStreetMap Contributors (2016)	44–1503	256	±213	
Area of surrounding forest	km^2^	Total size of the forest patch which contains the plot		OpenStreetMap Contributors (2016)	0.14–333.62	96.64	±112.97	
Forest connectivity	%	Percentage of forest cover in the 25 km^2^ surrounding the plot center		ConFoBi data	3.0–92.2	59.9	±19.7	
Edge density (10 ha surrounding plot center)	m/ha	Sum of lengths (m) of all edge segments involving forests per 1‐ha plot; mean value derived from landuse map (Landsat TM5; yrs 2009, 2010)		LUBW (2010)	121–350	226	±61	McGarigal (2015)
Euclidean nearest neighbor distance (20 ha), CV	m	Coefficient of variation of the distance (m) to the nearest neighboring patch of forest, based on shortest edge‐to‐edge distance; derived from landuse map (Landsat TM5; yrs 2009, 2010)		LUBW (2010)	0–42.5	9.8	±8.7	McGarigal (2015)
Euclidean nearest neighbor distance (20 ha), mean	m	Area‐weighted mean distance (m) to the nearest neighboring patch of forest, based on shortest edge‐to‐edge distance; derived from landuse map (Landsat TM5; yrs 2009, 2010)		LUBW (2010)	0–66.5	12.7	±12.0	McGarigal (2015)
Euclidean nearest neighbor distance (50 ha), mean	m	Area‐weighted mean distance (m) to the nearest neighboring patch of forest, based on shortest edge‐to‐edge distance; derived from landuse map (Landsat TM5; yrs 2009, 2010)		LUBW (2010)	1.56–152.6	70.7	35.5	McGarigal (2015)
Euclidean nearest neighbor distance (50 ha), CV	m	Coefficient of variation derived from landuse map (Landsat TM5; yrs 2009, 2010)		LUBW (2010)	0–51	9.4	±8.8	McGarigal (2015)
Aggregation index (50 ha)	%	Mean number of like adjacencies involving forest divided by the maximum possible number of like adjacencies involving forest; multiplied by 100. From landuse map (Landsat TM5; yrs 2009, 2010)		LUBW (2010)	64.8–99.5	83.1	±7.4	McGarigal (2015)
Contiguity index (50 ha)		The sum of the cells divided by the total number of pixels in the patch minus 1, divided by the sum of the template values minus 1. Area‐weighted mean derived from Landsat TM5 (yrs 2009, 2010)		LUBW (2010)	0.00167–0.02156	0.00875	±0.00459	McGarigal (2015)
Landscape shape index (50 ha)		0.25 the sum of entire landscape boundary and edge segments (m) within landscape boundary involving forest, divided by square root of total landscape area (m^2^). Mean derived from Landsat TM5 (yrs 2009, 2010)		LUBW (2010)	1.08–5.25	3.19	±0.91	McGarigal (2015)
Perimeter‐area ratio distribution (50 ha)	m/m^2^	A simple measure of shape complexity. Area‐weighted mean derived from Landsat TM5 (yrs 2009, 2010)		LUBW (2010)	1.6–27.8	11.1	±5.7	McGarigal (2015)
Percentage of like adjacencies (50 ha)	%	Percentage of cell adjacencies involving forest that are like adjacencies. Mean derived from Landsat TM5; yrs 2009, 2010		LUBW (2010)	58.7–95.0	76.6	±7.9	McGarigal (2015)
Contiguity index (100 ha)		The sum of the cells divided by the total number of pixels in the patch minus 1, divided by the sum of the template values minus 1. Area‐weighted mean derived from Landsat TM5 (yrs 2009, 2010)		LUBW (2010)	0.0007–0.0149	0.0057	±0.0032	McGarigal (2015)
Core area (100 ha)	m^2^	Area (m^2^) within the patch that is further than the specified depth‐of‐edge distance from the patch perimeter. Area‐weighted mean derived from landuse map (Landsat TM5; yrs 2009, 2010)		LUBW (2010)	0.06–5.11	1.44	±0.99	McGarigal (2015)
Splitting index (100 ha)		Total area (m^2^) squared divided by the sum of patch area (m^2^) squared, summed across all patches of forest. Mean derived from Landsat TM5; yrs 2009, 2010		LUBW (2010)	0.007–1.35	0.23	±0.25	McGarigal (2015)

## References

[ece36003-bib-0001] Aldinger, E. , Hübner, W. , Michiels, H.‐G. , Mühlhäußer, G. , Schreiner, M. , & Wiebel, M. (1998). Überarbeitung der standortskundlichen regionalen Gliederung im Südwestdeutschen Stand‐ortskundlichen Verfahren. Mitteilungen des Vereins für Forstliche Standortserkundung und Forstpflanzenzüchtung, 33, 9–26.

[ece36003-bib-0002] Asbeck, T. , Pyttel, P. , Frey, J. , & Bauhus, J. (2019). Predicting abundance and diversity of tree‐related microhabitats in Central European montane forests from common forest attributes. Forest Ecology and Management, 432, 400–408. 10.1016/j.foreco.2018.09.043

[ece36003-bib-0003] Augustynczik, A. L. D. , Asbeck, T. , Basile, M. , Bauhus, J. , Storch, I. , Mikusinski, G. , … Hanewinkel, M. (2019). Diversification of forest management regimes secures tree microhabitats and bird abundance under climate change. Science of the Total Environment, 650, 2717–2730. 10.1016/j.scitotenv.2018.09.366 30296777

[ece36003-bib-0004] Augustynczik, A. L. D. , Gutsch, M. , Basile, M. , Suckow, F. , Lasch, P. , Yousefpour, R. , & Hanewinkel, M. (2020). Socially optimal forest management and biodiversity conservation in temperate forests under climate change. Ecological Economics 169, 106504 10.1016/j.ecolecon.2019.106504

[ece36003-bib-0005] Augustynczik, A. L. D. , Hartig, F. , Minunno, F. , Kahle, H.‐P. , Diaconu, D. , Hanewinkel, M. , & Yousefpour, R. (2017). Productivity of Fagus sylvatica under climate change – A Bayesian analysis of risk and uncertainty using the model 3‐PG. Forest Ecology and Management, 401, 192–206. 10.1016/j.foreco.2017.06.061

[ece36003-bib-0006] Augustynczik, A. L. D. , Yousefpour, R. , & Hanewinkel, M. (2018). Multiple uncertainties require a change of conservation practices for saproxylic beetles in managed temperate forests. Scientific Reports, 8, 14964 10.1038/s41598-018-33389-9 30297782 PMC6175923

[ece36003-bib-0008] Augustynczik, A. L. D. , Yousefpour, R. , & Hanewinkel, M. (2019). Impacts of climate change on the supply of biodiversity in temperate forest landscapes. Allgemeine Forst Und Jagdzeitung 189(11/12), 209–220.

[ece36003-bib-0009] Augustynczik, A. L. D. , Yousefpour, R. , Rodriguez, L. C. E. , & Hanewinkel, M. (2018). Conservation costs of retention forestry and optimal habitat network selection in southwestern Germany. Ecological Economics, 148, 92–102. 10.1016/j.ecolecon.2018.02.013

[ece36003-bib-0010] Baeten, L. , Verheyen, K. , Wirth, C. , Bruelheide, H. , Bussotti, F. , Finér, L. , … Scherer‐Lorenzen, M. (2013). A novel comparative research platform designed to determine the functional significance of tree species diversity in European forests. Perspectives in Plant Ecology, 15, 281–291. 10.1016/j.ppees.2013.07.002

[ece36003-bib-0011] Basile, M. , Mikusinski, G. , & Storch, I. (2019). Bird guilds show different responses to tree retention levels: A meta‐analysis. Global Ecology and Conservation, 18, e00615 10.1016/j.gecco.2019.e00615

[ece36003-bib-0012] Basile, M. , Mikusinski, G. , & Storch, I. (2020). Woodpecker cavity establishment in managed forests: Relative rather than absolute tree size matters. Wildlife Biology. 10.2981/wlb.00564

[ece36003-bib-0013] Bauhus, J. , Puettmann, K. J. , & Kuehne, C. (2013). Is close‐to‐nature forest management in Europe compatible with managing forests as complex adaptive forest ecosystems? In Messier C., Puettmann K. J., & Coates K. D. (Eds.), Managing forests as complex adaptive systems: Building resilience to the challenge of global change (pp. 187–213) (1st ed.). Oxon, UK: Routledge.

[ece36003-bib-0014] Bauhus, J. , Puettmann, K. , & Messier, C. (2009). Silviculture for old‐growth attributes. Forest Ecology and Management, 258, 525–537. 10.1016/j.foreco.2009.01.053

[ece36003-bib-0015] Bauhus, J. , & Pyttel, P. (2015). Managed forests In Peh K.‐ S.‐H., Corlett R. T., & Bergeron Y. (Eds.) Routledge handbook of forest ecology (pp. 75–90) (1st ed.). Oxon, UK: Routledge.

[ece36003-bib-0016] BaySF . (2009). Naturschutzkonzept der Bayerischen Staatsforsten. Regensburg: Bayerische Staatsforsten (BaySF) AöR.

[ece36003-bib-0017] Bennett, N. J. , Roth, R. , Klain, S. C. , Chan, K. , Christie, P. , Clark, D. A. , … Wyborn, C. (2017). Conservation social science: Understanding and integrating human dimensions to improve conservation. Biological Conservation, 205, 93–108. 10.1016/j.biocon.2016.10.006

[ece36003-bib-0018] BMU . (2007). National Stretegy on Biodiversity. Nature Conservation and Nuclear Safety (BMU), Berlin: Federal Ministry for the Environment https://biologischevielfalt.bfn.de/fileadmin/NBS/documents/Veroeffentlichungen/BMU_Natio_Strategie_en_bf.pdf

[ece36003-bib-0019] Bollmann, K. , & Braunisch, V. (2013). To integrate or to segregate: Balancing commodity production and biodiversity conservation in European forests In Kraus D., & Krumm F. (Eds.), Integrative approaches as an opportunity for the conservation of forest biodiversity (pp. 18–31). Joensuu, Finland: European Forest Institute.

[ece36003-bib-0020] Bujoczek, L. , Szewczyk, J. , & Bujoczek, M. (2018). Deadwood volume in strictly protected, natural and primeval forests in Poland. European Journal of Forest Research, 137, 401–418. 10.1007/s10342-018-1124-1

[ece36003-bib-0021] Bütler, R. , Lachat, T. , Larrieu, L. , & Paillet, Y. (2013). Habitat trees: Key elements for forest biodiversity In Kraus D., & Krumm F. (Eds.), Integrative approaches as an opportunity for the conservation of forest biodiversity (pp. 84–91). Joensuu, Finland: European Forest Institute.

[ece36003-bib-0022] ESA . (2018). Sentinel 2 data, Copernicus Open Access Hub of the European Space Agency (ESA). Retrieved from https://www.scihub.copernicus.eu/

[ece36003-bib-0023] European Commission . (2011). Our life insurance, our natural capital: An EU biodiversity strategy to 2020. Brussels, Belgium: Communication from the Commission to the European Parliament, the Council, the European Economic and Social Committee and Committee of the Regions (COM 2011/244 final).

[ece36003-bib-0024] European Commission . (2013). A new EU forest strategy: For forests and the forest‐based sector. Brussels, Belgium: Communication from the Commission to the European Parliament, the Council, the European Economic and Social Committee and Committee of the Regions (COM 2013/659 final).

[ece36003-bib-0025] European Environment Agency (EEA) . (2016). European forest ecosystems, state and trends. EEA‐report 5/2016. Luxembourg, Luxembourg: Publications Office of the European Union 10.2800/964893

[ece36003-bib-0026] Federal Ministry of Food, Agriculture and Consumer Protection (BMELV) . (2011). Forest strategy 2020, sustainable forest management – An opportunity and a challenge for society. Bonn, Germany: BMELV.

[ece36003-bib-0027] Fedrowitz, K. , Koricheva, J. , Baker, S. C. , Lindenmayer, D. B. , Palik, B. , Rosenvald, R. , … Gustafsson, L. (2014). Can retention forestry help conserve biodiversity? A meta‐analysis. Journal of Applied Ecology, 51, 1669–1679. 10.1111/1365-2664.12289 25552747 PMC4277688

[ece36003-bib-0028] Fischer, M. , Bossdorf, O. , Gockel, S. , Hänsel, F. , Hemp, A. , Hessenmöller, D. , … Weisser, W. W. (2010). Implementing large‐scale and long‐term functional biodiversity research: The biodiversity exploratories. Basic and Applied Ecology, 11, 473–485. 10.1016/j.baae.2010.07.009

[ece36003-bib-0029] FoGIS10/InFoGIS . (2018). Stand based forest inventory of the State of Baden‐Württemberg, MLR, Stuttgart. Retrieved from https://www.sta-uis.de/Systembeschreibungen-Baden-Wuerttemberg-Forstliches-Geographisches-Informationssystem.html

[ece36003-bib-0030] Forest Europe . (2015). State of Europe's forests 2015. Madrid, Spain: Forest Europe Retrieved from http://www.foresteurope.org/docs/fullsoef2015.pdf

[ece36003-bib-0032] ForstBW . (2015). Die Gesamtkonzeption Waldnaturschutz ForstBW, mit den Waldnaturschutzzielen 2020. Stuttgart, Germany: Landesbetrieb ForstBW.

[ece36003-bib-0033] ForstBW . (2016). Alt‐ und Totholz‐Konzept Baden‐Württemberg. Stuttgart, Germany: Landesbetrieb ForstBW.

[ece36003-bib-0034] Franklin, C. M. A. , Macdonald, S. E. , & Nielsen, S. E. (2019). Can retention harvests help conserve wildlife? Evidence for vertebrates in the boreal forest. Ecosphere, 10, e02632 10.1002/ecs2.2632

[ece36003-bib-0035] Frey, J. , Joa, B. , Schraml, U. , & Koch, B. (2019). Same viewpoint different perspectives—A comparison of expert ratings with a TLS derived forest stand structural complexity index. Remote Sensing, 11, 1137 10.3390/rs11091137

[ece36003-bib-0036] Frey, J. , Kovach, K. , Stemmler, S. , & Koch, B. (2018). UAV photogrammetry of forests as a vulnerable process. A sensitivity analysis for a structure from motion RGB‐image pipeline. Remote Sensing, 10, 912 10.3390/rs10060912

[ece36003-bib-0037] Gauer, J. , & Aldinger, E. (2005). Waldökologische Naturräume Deutschlands – Forstliche Wuchsgebiete und Wuchsbezirke mit Karte im Maßstab 1:1000.000. Freiburg, Germany: Verein für Forstliche Standortkunde und Forstpflanzenzüchtung.

[ece36003-bib-0038] Goldberg, E. , Kirby, K. , Hall, J. , & Latham, J. (2007). The ancient woodland concept as a practical conservation tool in Great Britain. Journal for Nature Conservation, 15, 109–119. 10.1016/j.jnc.2007.04.001

[ece36003-bib-0039] Gorenflo, L. J. , & Brandon, K. (2006). Key human dimensions of gaps in global biodiversity conservation. BioScience, 56, 723–731. 10.1641/0006-3568(2006)56[723:KHDOGI]2.0.CO;2

[ece36003-bib-0040] Gustafsson, L. , Baker, S. C. , Bauhus, J. , Beese, W. J. , Brodie, A. , Kouki, J. , … Franklin, J. F. (2012). Retention forestry to maintain multifunctional forests: A world perspective. BioScience, 62, 633–645. 10.1525/bio.2012.62.7.6

[ece36003-bib-0041] Gustafsson, L. , Bauhus, J. , Asbeck, T. , Augustynczik, A. L. D. , Basile, M. , Frey, J. , … Storch, I. (2019). Retention as an integrated biodiversity conservation approach for continuous‐cover forestry in Europe. Ambio, 49, 85–97. 10.1007/s13280-019-01190-1 31055795 PMC6889099

[ece36003-bib-0042] Gutzat, F. , & Dormann, C. F. (2018). Decaying trees improve nesting opportunities for cavity‐nesting birds in temperate and boreal forests: A meta‐analysis and implications for retention forestry. Ecology and Evolution, 8, 8616–8626. 10.1002/ece3.4245 30250728 PMC6144968

[ece36003-bib-0043] Hilmers, T. , Friess, N. , Bässler, C. , Heurich, M. , Brandl, R. , Pretzsch, H. , … Müller, J. (2018). Biodiversity along temperate forest succession. Journal of Applied Ecology, 55, 2756–2766. 10.1111/1365-2664.13238

[ece36003-bib-0044] IPBES . (2019). Summary for policymakers of the global assessment report on biodiversity and ecosystem services of the Intergovernmental Science‐Policy Platform on Biodiversity and Ecosystem Services. Díaz S., Settele J., Brondízio E. S., Ngo H. T., Guèze M., Agard J., Arneth A., Balvanera P., Brauman K. A., Butchart S. H. M., Chan K. M. A., Garibaldi L. A., Ichii K., Liu J., Subramanian S. M., Midgley G. F., Miloslavich P., Molnár Z., Obura D., Pfaff A., Polasky S., Purvis A., Razzaque J., Reyers B., Roy Chowdhury R., Shin Y. J., Visseren-Hamakers I. J., Willis K. J., & Zayas C. N. (Eds.). Bonn, Germany: IPBES Secretariat 10.5281/zenodo.3553579

[ece36003-bib-0045] Joa, B. , & Schraml, U. (2018). Die Bedeutung lokalen ökologischen Wissens für den Erhalt der Waldbiodiversität In Korn H., & Dünnfelder H. (Eds.), Treffpunkt Biologische Vielfalt XVII ‐ Interdisziplinärer Forschungsaustausch im Rahmen des Übereinkommens über biologische Vielfalt (pp. 149–157). Bonn: Bundesamt für Naturschutz (BfN). BfN Skripten 527.

[ece36003-bib-0046] Joa, B. , Winkel, G. , & Primmer, E. (2018). The unknown known – A review of local ecological knowledge in relation to forest biodiversity conservation. Land Use Policy, 79, 520–530. 10.1016/j.landusepol.2018.09.001

[ece36003-bib-0047] Kahl, T. , & Bauhus, J. (2014). An index of forest management intensity based on assessment of harvested tree volume, tree species composition and dead wood origin. Nature Conservation, 7, 15–27. 10.3897/natureconservation.7.7281

[ece36003-bib-0048] Kändler, G. , & Cullmann, D. (2014). Der Wald in Baden‐Württemberg – Ausgewählte Ergebnisse der dritten Bundeswaldinventur. Freiburg, Germany: Forstliche Versuchs‐ und Forschungsanstalt Baden‐Württemberg (FVA).

[ece36003-bib-0049] Kändler, G. , & Cullmann, D. (2016). Regionale Auswertung der Bundeswaldinventur 3, Wuchsgebiet Schwarzwald, Freiburg. Freiburg, Germany: Forstliche Versuchs‐ und Forschungsanstalt Baden‐Württemberg (FVA).

[ece36003-bib-0050] Knuff, A. K. , Staab, M. , Frey, J. , Helbach, J. , & Klein, A. M. (2019). Plant composition, not richness, drives occurrence of specialist hervivores. Ecological Entomology, 44, 833–843. 10.1111/een.12767

[ece36003-bib-0051] Knuff, A. K. , Winiger, N. , Klein, A. M. , Segelbacher, G. , & Staab, M. (2019). Optimising sampling of flying insects using a modified window trap. Methods in Ecology and Evolution, 10, 1820–1825. 10.1111/2041-210X.13258

[ece36003-bib-0053] Kraus, D. , & Krumm, F. (2013). Integrative approaches as an opportunity for the conservation of forest biodiversity. Joensuu, Finland: European Forest Institute.

[ece36003-bib-0055] Larrieu, L. , Paillet, Y. , Winter, S. , Bütler, R. , Kraus, D. , Krumm, F. , … Vandekerkhove, K. (2017). Tree related microhabitats in temperate and Mediterranean European forests: A hierarchical typology for inventory standardization. Ecological Indicators, 84, 194–207. 10.1016/j.ecolind.2017.08.051

[ece36003-bib-0056] Leibold, M. A. , Holyoak, M. , Mouquet, N. , Amarasekare, P. , Chase, J. M. , Hoopes, M. F. , … Gonzalez, A. (2004). The metacommunity concept: A framework for multi‐scale community ecology. Ecology Letters, 7, 601–613. 10.1111/j.1461-0248.2004.00608.x

[ece36003-bib-0057] LGL . (2005). Digital Terrain Model. Landesamt für Geoinformation und Landentwicklung of the State of Baden‐Württemberg, Stuttgart. Retrieved from https://www.lgl-bw.de/lgl-internet/opencms/de/05_Geoinformation/Geotopographie/Digitale_Gelaendemodelle/

[ece36003-bib-0058] Lindenmayer, D. B. , & Franklin, J. F. (2002). Conserving forest biodiversity: A comprehensive multiscaled approach. Washington, DC: Island Press.

[ece36003-bib-0059] LUBW . (2010). Land Cover derived from Landsat 2010 data. Stuttgart, Germany: Landesanstalt für Umwelt Baden‐Württemberg, and Ministerium für Umwelt, Klima und Energiewirtschaft Baden‐Württemberg Retrieved from https://rips-dienste.lubw.baden-wuerttemberg.de/rips/ripsservices/apps/uis/metadaten/beschreibung.aspx?typ=0%26uuxml:id=db9d1dd9-158a-4b6e-b9a0-701330242b90

[ece36003-bib-0060] Maier, C. , & Winkel, G. (2017). Implementing nature conservation through integrated forest management: A street‐level bureaucracy perspective on the German public forest sector. Forest Policy and Economics, 82, 14–29. 10.1016/j.forpol.2016.12.015

[ece36003-bib-0061] McGarigal, K. (2015). Fragstats help. Retrieved from https://www.umass.edu/landeco/research/fragstats/fragstats.html

[ece36003-bib-0062] Mehring, M. , Bernard, B. , Hummel, D. , Liehr, S. , & Lux, A. (2017). Halting biodiversity loss: How social‐ecological biodiversity research makes a difference. International Journal of Biodiversity Sciences Ecosystem Services & Management, 13, 172–180. 10.1080/21513732.2017.1289246

[ece36003-bib-0063] Mönkkönnen, M. , Ylisirniö, A.‐L. , & Hämäläinen, T. (2009). Ecological efficiency of voluntary conservation of boreal‐forest biodiversity. Conservation Biology, 23, 339–347. 10.1111/j.1523-1739.2008.01082.x 18983601

[ece36003-bib-0064] Mori, A. S. , Tatsumi, S. , & Gustafsson, L. (2017). Landscape properties affect biodiversity response to retention approaches in forestry. Journal of Applied Ecology, 54, 1627–1637. 10.1111/1365-2664.12888

[ece36003-bib-0065] Müller, J. (2005). Waldstrukturen als Steuergröße für Artengemeinschaften in kollinen bis submontanen Buchenwäldern. Dissertation, Technische Universität München.

[ece36003-bib-0066] Müller, J. , & Bütler, R. (2010). A review of habitat thresholds for dead wood: A baseline for management recommendations in European forests. European Journal of Forest Research, 129, 981–992. 10.1007/s10342-010-0400-5

[ece36003-bib-0067] Musacchio, L. R. (2009). The scientific basis for the design of landscape sustainability: A conceptual framework for translational landscape research and practice of designed landscapes and the six Es of landscape sustainability. Landscape Ecology, 24, 993–1013. 10.1007/s10980-009-9396-y

[ece36003-bib-0068] Niemelä, J. , Young, J. , Alard, D. , Askasibar, M. , Henle, K. , Johnson, R. , … Watt, A. (2005). Identifying, managing and monitoring conflicts between forest biodiversity conservation and other human interests in Europe. Forest Policy and Economics, 7, 877–890. 10.1016/j.forpol.2004.04.005

[ece36003-bib-0069] OpenStreetMap Contributors . (2016). CC BY‐SA. Retrieved from https://www.openstreetmap.org/copyright

[ece36003-bib-0070] Paillet, Y. , Archaux, F. , du Puy, S. , Bouget, C. , Boulanger, V. , Debaive, N. , … Guilbert, E. (2018). The indicator side of tree microhabitats: A multi‐taxon approach based on bats, birds and saproxylic beetles. Journal of Applied Ecology, 55, 2147–2159. 10.1111/1365-2664.13181

[ece36003-bib-0071] Percel, G. , Laroche, F. , & Bouget, C. (2019). The scale of saproxylic beetles response to landscapestructure depends on their habitat stability. Landscape Ecology 34, 1905–1918. 10.1007/s10980-019-00857

[ece36003-bib-0072] Pregernig, M. (2014). Framings of science‐policy interactions and their discursive and institutional effects: Examples from conservation and environmental policy. Biodiversity and Conservation, 23, 3615–3639. 10.1007/s10531-014-0806-3

[ece36003-bib-0073] Ranius, T. , & Fahrig, L. (2006). Targets for maintenance of dead wood for biodiversity conservation based on extinction thresholds. Scandinavian Journal of Forest Research, 21, 201–208. 10.1080/02827580600688269

[ece36003-bib-0074] Rosenkranz, L. , Seintsch, B. , Wippel, B. , & Dieter, M. (2014). Income losses due to the implementation of the habitats directive in forests—Conclusions from a case study in Germany. Forest Policy and Economics, 38, 207–218. 10.1016/j.forpol.2013.10.005

[ece36003-bib-0075] Rouse, J. W. , Haas, R. H. , Schell, J. A. , & Deering, W. D. (1973). Monitoring vegetation systems in the Great Plains with ERTS. Third ERTS symposium, NASA SP‐351 (pp. 309–317).

[ece36003-bib-0076] Rutte, C. (2011). The sacred commons: Conflicts and solutions of resource management in sacred natural sites. Biological Conservation, 144, 2387–2394. 10.1016/j.biocon.2011.06.017

[ece36003-bib-0077] Sandström, J. M. , Bernes, C. , Junninen, K. , Lõhmus, A. , Macdonald, E. , Müller, J. , & Jonsson, B. G. (2019). Impacts of dead‐wood manipulation on the biodiversity of temperate and boreal forests: A systematic review. Journal of Applied Ecology, 56, 1770–1781. 10.1111/1365-2664.13395

[ece36003-bib-0078] Scherzinger, W. (1996). Naturschutz im Wald: Qualitätsziele einer dynamischen Waldentwicklung. Praktischer Naturschutz. Stuttgart, Germany: Verlag Eugen Ulmer 10.1002/mmnz.19980740118

[ece36003-bib-0079] Schindler, S. , von Wehrden, H. , Poirazidis, K. , Wrbka, T. , & Kati, V. (2013). Multiscale performance of landscape metrics as indicators of species richness of plants, insects and vertebrates. Ecological Indicators, 31, 41–48. 10.1016/j.ecolind.2012.04.012

[ece36003-bib-0080] Schlesinger, W. H. (2010). Translational ecology. Science, 329, 609 10.1126/science.1195624 20688985

[ece36003-bib-0081] Siitonen, J. , Martikainen, P. , Punttila, P. , & Rauh, J. (2000). Coarse woody debris and stand characteristics in mature managed and old‐growth boreal mesic forests in southern Finland. Forest Ecology and Management, 128, 211–225. 10.1016/S0378-1127(99)00148-6

[ece36003-bib-0082] Sotirov, M. (Ed.) (2017). Natura 2000 and forests: Assessing the state of implementation and effectiveness. What science can tell us 7, Joensuu, Finland: European Forest Institute.

[ece36003-bib-0085] Tinch, R. , Balian, E. , Carss, D. , Ezzine‐de‐Blas, D. , Geamana, N. A. , Heink, U. , … Young, J. C. (2018). Science‐policy interfaces for biodiversity: Dynamic learning environments for successful impact. Biodiversity and Conservation, 27, 1679–1702. 10.1007/s10531-016-1155-1

[ece36003-bib-0086] Tscharntke, T. , Tylianakis, J. M. , Rand, T. A. , Didham, R. K. , Fahrig, L. , Batáry, P. , … Westphal, C. (2012). Landscape moderation of biodiversity patterns and processes – Eight hypotheses. Biological Reviews, 87, 661–685. 10.1111/j.1469-185X.2011.00216.x 22272640

[ece36003-bib-0087] UNECE/FAO . (2000). Forest resources of Europe, CIS, North America, Australia, Japan and New Zealand (TBFRA 2000). Main report. UNECE/FAO Contribution to the Global Forest Resources Assessment 2000. New York, NY and Geneva, Switzerland: United Nations.

[ece36003-bib-0088] Valdés, A. , Lenoir, J. , Gallet‐Moron, E. , Andrieu, E. , Brunet, J. , Chabrerie, O. , … Decocq, G. (2015). The contribution of patch‐scale conditions is greater than that of macroclimate in explaining local plant diversity in fragmented forests across Europe. Global Ecology and Biogeography, 24, 1094–1105. 10.1111/geb.12345

[ece36003-bib-0089] Van Wagner, C. E. (1982). Practical aspects of the line intersect method. Canadian Forestry Service, information report PI‐X‐12. Retrieved from https://cfs.nrcan.gc.ca/publications?xml:id=6862

[ece36003-bib-0090] Verkerk, P. J. , Mavsar, R. , Giergiczny, M. , Lindner, M. , Edwards, D. , & Schelhaas, M. J. (2014). Assessing impacts of intensified biomass production and biodiversity protection on ecosystem services provided by European forests. Ecosystem Services, 9, 155–165. 10.1016/j.ecoser.2014.06.004

[ece36003-bib-0091] Vítková, L. , Bače, R. , Kjučukov, P. , & Svoboda, M. (2018). Deadwood management in Central European forests: Key considerations for practical implementation. Forest Ecology and Management, 429, 394–405. 10.1016/j.foreco.2018.07.034

[ece36003-bib-0092] Wilson, M. F. J. , O'Connell, B. , Brown, C. , Guinan, J. C. , & Grehan, A. J. (2007). Multiscale terrain analysis of multibeam bathymetry data for habitat mapping on the continental slope. Marine Geodesy, 30, 3–35. 10.1080/01490410701295962

[ece36003-bib-0093] Winter, S. , Borrass, L. , Geitzenauer, M. , Blondet, M. , Breibeck, R. , Weiß, G. , & Winkel, G. (2014). The impact of natura 2000 on forest management – A socio‐ecological analysis in the continental region of the European Union. Biodiversity and Conservation, 23, 3451–3482. 10.1007/s10531-014-0822-3

[ece36003-bib-0094] Wirth, V. , Tønsberg, T. , Reif, A. , & Stevenson, D. (2018). Loxospora Cristinae Found in Germany. Herzogia, 31, 995–999. 10.13158/heia.31.2.2018.995

